# Risk perceptions of individuals living in single-parent households during the COVID-19 crisis: examining the mediating and moderating role of income

**DOI:** 10.3389/fsoc.2023.1265302

**Published:** 2023-11-30

**Authors:** Bernd Liedl, Nina-Sophie Fritsch, Cristina Samper Mejia, Roland Verwiebe

**Affiliations:** ^1^Department of Sociology, University of Vienna, Vienna, Austria; ^2^Institute for Sociology and Social Research, Vienna University of Economics and Business, Vienna, Austria; ^3^Faculty of Economics and Social Science, University of Potsdam, Potsdam, Germany

**Keywords:** individuals living in single-parent households, household types, COVID-19 pandemic, subjective risk perception, objective labour market outcome, Germany

## Abstract

The COVID-19 crisis had severe social and economic impact on the life of most citizens around the globe. Individuals living in single-parent households were particularly at risk, revealing detrimental labour market outcomes and assessments of future perspectives marked by worries. As it has not been investigated yet, in this paper we study, how their perception about the future and their outlook on how the pandemic will affect them is related to their objective economic resources. Against this background, we examine the subjective risk perception of worsening living standards of individuals living in single-parent households compared to other household types, their objective economic situation based on the logarithmised equivalised disposable household incomes and analyse the relationship between those indicators. Using the German SOEP, including the SOEP-CoV survey from 2020, our findings based on regression modelling reveal that individuals living in single-parent households have been worse off during the pandemic, facing high economic insecurity. Path and interaction models support our assumption that the association between those indicators may not be that straightforward, as there are underlying mechanisms–such as mediation and moderation–of income affecting its direction and strength. With respect to our central hypotheses, our empirical findings point toward (1) a mediation effect, by demonstrating that the subjective risk perception of single-parent households can be partly explained by economic conditions. (2) The moderating effect suggests that the concrete position at the income distribution of households matters as well. While at the lower end of the income distribution, single-parent households reveal particularly worse risk perceptions during the pandemic, at the high end of the income spectrum, risk perceptions are similar for all household types. Thus, individuals living in single-parent households do not perceive higher risks of worsening living standards due to their household situation *per se*, but rather because they are worse off in terms of their economic situation compared to individuals living in other household types.

## 1 Introduction

The COVID-19 pandemic had enormous social and economic impact on individuals around the globe. Millions of people were severely affected in terms of their health (Kontoangelos et al., 2020); massively restricted in their personal freedom (e.g., social distancing and lockdowns) (Tisdell, 2020); had to make major changes to their daily routines (Broersma, 2022; Li et al., 2022); remained numerous weeks in furlough with minimal income, or even gradually stumbled into unemployment after dramatically reduced working hours (Schulten and Müller, 2020). In Germany, despite multiple policy interventions intended to protect citizens from infection as well as from economic hardship, severe consequences on the life of most residents could not be forestalled. More than 13.5 million individuals’ incomes fell below the poverty line in 2021, which presents an all-time high of poverty rates in Germany; unemployment rates also escalated severely (Schneider et al., 2022). Job and/or income loss during this crisis also bear the risk of increasing socioeconomic stress for individuals. This can lead to impaired risk perception and lower subjective well-being, even resulting in anxiety and/or depression (Fancourt et al., 2020; Ettman et al., 2021). As the pandemic prolonged, risk perceptions, states of mental health and well-being were likewise deteriorating (Entringer and Kröger, 2021; Hiekel and Kühn, 2022; Romero-Gonzalez et al., 2022). Thus, the severe consequences of the COVID-19 crisis can be displayed by a set of objective (e.g., income, unemployment rate, hours employed, number of days of sick leave) and subjective (e.g., risk perception, well-being, life satisfaction) indicators.

Looking at some of these indicators separately, prior research has shown that the recent crisis has hit certain social groups harder than others and that socioeconomic risks are not equally distributed among different household types in Germany (BMAS, 2017; Butterwege, 2021; Hipp and Bünning, 2021; Huebener et al., 2021; Kreyenfeld and Zinn, 2021; Kuhn et al., 2021; Blundell et al., 2022; Li et al., 2022). In a nutshell, those indicators emphasise that individuals living in single-parent households^1^ were particularly affected during the pandemic (Dromey et al., 2020; Hertz et al., 2021). They faced the highest poverty rate of all household types (42 percent in 2022) and revealed worrying perceptions about their future (Schneider et al., 2022). This may not come as a surprise since they had to manage additional obstacles such as an increased burden of unpaid housework and home schooling overnight. Single parents were severely affected by the shift of all childcare responsibilities from formal institutions to private households, putting them under enormous stress and only raising more concerns about caregivers’ mental health and wellbeing (Li et al., 2022). Where couple-parent households with children at least had greater flexibility arranging their additional tasks and time budgets for balancing work and family issues, single parents did not even have the comforting support of a partner. In fact, single parents had to shoulder it all on their own and were left alone to cope with the impossible in times of increasing uncertainty (O'Reilly, 2020; Carotta et al., 2022).

Interestingly enough, previous research on the situation of single-parent households during the COVID-19 crisis has not sufficiently investigated the relationship between those subjective and objective indicators so far. It is still not entirely clear how to explain their perception about the future and to what extent their outlook is related to their objective economic conditions. In order to close this research gap, we analyse their situation during the pandemic, by determining how new (and worsened) economic realities influence the subjective future risk perception of individuals living in single-parent households.

Against this background, we go beyond previous research as we do not only examine (i) the subjective indicator of individual risk perception of individuals living in single-parent households and (ii) their objective economic situation (based on the logarithm of their equivalised disposable household income), but (iii) also assess the relationship between those indicators. In applying this approach, we focus on the experiences of individuals living in single-parent households in Germany during the pandemic, while comparing them to individuals living in three other household types (singles without children, couple-parent households with children, couples without children). For our analyses, we use data from the German Socio-Economic Panel (GSOEP), including the specific SOEP-CoV survey from 2020, which observed the same individuals before and during the COVID-19 crisis. As our modelling strategy, we apply path and interaction models (Baron and Kenny, 1986; Aichholzer, 2017) in order to disentangle the seemingly obvious relation between household type and risk perception, whilst considering income as mediating and moderating variable. Here, path modelling is particularly suitable to test a mediation relation, because (1) it allows us to look at the relationship of two variables (in our case household type and risk perception) at the same time, next to (2) analysing the changing relation between them once we include another explanatory variable (income). In addition, an interaction model (between household type and income) allows us to test whether and to what extent the income level affects the risk perceptions of different household types.

Our findings reveal that individuals living in single-parent households have been worse off in the past decades and continue to be a special risk group, showing high economic insecurity during the pandemic. Although individuals in different household types seem to reveal unequal risk perceptions at first glance, these effects forfeit explanatory power once we include income into the model. In particular, we find that their economic situation mediates the effect of household types on risk perception during the COVID-19 crisis. Furthermore, the interaction model reveals that the level of income does moderate the risk perception of parents in contrast to non-parents, yet both partnered parents and single parents share similar negative risk perceptions when they earn a low income. Since single-parent households are likely to have a low income, this also largely explains the differences in risk perceptions between coupled parents and single parents. Thus, our findings demonstrate that the weak financial situation prevalent amongst individuals living in single parent households is inherent in their comparably more negative future risk perception.

## 2 Background information on individuals living in single-parent households in Germany

Providing some institutional background information, Germany is categorised as a corporatist welfare state, coinciding with a (modernised) male-breadwinner model/female caregiver model, shaping the distribution of resources and opportunities contingent on employment or family position (Esping-Andersen, 1990; Lewis, 1992; Orloff, 1996; Lohmann and Zagel, 2016). For most of the time after the German reunification, family and social policies favoured traditional couple-parent households through the tax code, health insurance, child care, child benefits and other social security regulations, thus either perpetuating women’s dependence on a male breadwinner or disadvantaging single-parent households (Trappe et al., 2015, p. 232). In the course of an expanding service sector, however, the female employment rate increased steadily (even if almost always in part-time work), in turn fostering women’s labour market attachment, their educational attainment and progressive gender role norms (Brückner, 2004; Fritsch, 2014; Fritsch et al., 2022). In line with these changing contextual conditions and combined with an ongoing flexibilization of the labour market addressing an overall economic crisis in the 1990’s (Verwiebe and Fritsch, 2011; Verwiebe et al., 2013, 2014, 2019; Teitzer et al., 2014; Fritsch and Verwiebe, 2018; Riederer et al., 2019), Germany is slowly experiencing social policy changes (Streek, 2009; Hinrichs, 2010). This includes familialising policies such as the introduction of an earnings-related and gender-neutral parental leave benefit for the duration of 12–14 months, alongside de-familialising policies such as the expansion of childcare provision for children between the ages of one to 3 years, and a legal claim for publicly provided or subsidised childcare for every child over the age of one since 2013 (Seeleib-Kaiser, 2016, p. 225).

During the pandemic crisis, existing social security programs were substantially expanded and provided generous subsidies for German citizens. Especially with the social insurance program *Kurzarbeit* (short-time), authorities devised a massive 700 billion Euros plan, in order to protect worker’s income and prevent mass-layoffs; here, the government pays employees at least 60% of their regular pay for the hours not worked; and even 67% for working parents (Bariola and Collins, 2021, p. 1682). On the downside, this program does not include temporary and marginal employment, such as “mini jobs” for example. And indeed, single parents are more often in *Kurzarbeit* (short-time) and thus have to face above average income loss during the pandemic (BMFSFJ, 2021b, p. 26). Although the German welfare state is intended to promote social protection for vulnerable groups, we observe significant imbalances in terms of guaranteeing achieved living standards–especially for individuals living in single-parent households.

In order to build a bridge between institutional arrangements and economic realities of individuals living in different household types in Germany, we present some descriptive trends of how their shares have developed over the last two decades and portray their economic situation (median incomes, poverty risks, and unemployment rates) in Table 1 (the percentages are displayed for individuals who live in different household types). In Germany around 7.6 percent of the individuals live in single-parent households, and one of five households with children are headed by single parents, which corresponds to 6% of all households. Dependent children are living in around 1.5 million single-parent households, numbers that have stayed constant since 2009, 88% of them headed by females (BMFSFJ, 2021a).

**Table 1:** Economic situation of individuals living in different household types in Germany (2010–2020).

	Single-Parent households				Singles without children				Couple-Parent households				Couples without children				Total		
	% of Individuals	Unemployment	Risk of poverty	Median income	% of individuals	Unemployment	Risk of poverty	Median income	% of individuals	Unemployment	Risk of poverty	Median income	% of individuals	Unemployment	Risk of poverty	Median income	Unemployment	Risk of poverty	Median income
2000	6.3	12.2	23.7	920	17.4	9.0	14.4	1,125	46.8	6.8	9.2	1,128	26.7	9.3	5.7	1,363	8.3	10.2	1,166
2005	7.7	23.6	28.4	925	18.7	13.4	16.7	1,230	44.0	10.6	10.3	1,243	27.5	10.3	6.3	1,467	12.0	11.9	1,257
2010	7.8	21.4	32.5	982	20.7	12.4	18.1	1,300	41.3	7.5	10.2	1,400	28.9	6.5	6.9	1,667	9.4	12.7	1,400
2015	7.5	17.9	34.0	1,107	21.3	13.8	23.0	1,421	39.0	5.4	11.7	1,688	29.5	5.0	7.1	1,933	8.2	14.6	1,656
2019	7.3	15.2	34.4	1,333	21.6	10.7	24.7	1,600	39.8	4.9	12.3	1,923	28.8	4.4	8.4	2,267	6.9	15.5	1,900
2020	7.6	14.3	30.5	1,467	20.7	10.6	24.6	1,660	40.4	5.2	12.5	2,000	28.6	4.2	8.3	2,333	6.8	15.3	2,000

Source: GSOEP 2000–2020; own calculations for individuals aged 16–65, weighted results; percentages displayed for individuals living in different household types (single-parent households, singles without children, couple-parent households, couples without children).

The median income is based on the equivalised disposable household income for each individual living in the particular household type. Income is defined as the total income of a household, after tax and other deductions, that is available for spending or saving, divided by the number of household members converted into equalised adults; household members are equalised or made equivalent by weighting each according to their age, using the so-called modified OECD equivalence scale (Eurostat, 2021). Risk of poverty is defined as the share of individuals with an equivalised disposable income (after social transfer) below the at-risk-of-poverty threshold, which is set at 60% of the national median equivalised disposable income after social transfers. Unemployment rate is defined as the share of individuals aged 16–65 not employed during the reference week (Eurostat, 2021).

Furthermore, Table 1 reveals that economic risks are not equally distributed among individuals living in different household types and they are gaining relevance over the past decades in Germany (BMAS, 2017; Boehle, 2019; Schneider et al., 2022). This increase in inequality between different household types can be (at least partially) attributed to the massive labour market reforms (Hartz legislation). Overall, we observe that of all household types, single parents and their children are most often affected by socioeconomic risks, which only have become more pronounced in the past decades (Kraus, 2014). Within the last decades, we observe (1) a general tendency of decreasing unemployment rates, followed by (2) by increased poverty rates, which are (3) especially elevated for individuals living in single-parent households. With regard to the income development, it is apparent, that the median monthly household income of the total population has risen significantly in the last decade. However, this is not the case for individuals living in single-parents households. Rather, the income gap of individuals living in single-parent households has grown compared to the total German population.^2^

It is important to notice that in times of the COVID-19 pandemic, individuals living in single-parent households were worse off once again. Next to individuals living in households with three or more children (32 percent), single-parent households face the highest poverty rate of all household types in Germany in 2022 (42 percent) (Schneider et al., 2022). Federal intervention programs were likely to fizzle out due to high inflation rates and especially support households with proportionally higher incomes.^3^ Noticeable financial relief increases with the amount of income while the poorest again only received support insufficiently. Thus, the pandemic–followed by historically high levels of inflation–has widened the gap between poorer and richer households in Germany.

## 3 Individuals living in single-parent households, subjective risk perceptions, and income: prior and present research

### 3.1 Subjective indicator: individual risk perception of worsening living standards

As the subjective indicator we use the individual risk perception of worsening living standards. The concept of risk perception is complex and scholars from varying disciplines approach it differently, accounting for diverse ways in which people perceive and process risks they face in the social context of day-to-day life (Zinn, 2006; Soiné et al., 2021). One common denominator is the distinction between reality and possibility, where an undesirable state of reality^4^ may occur as a result of human activities or natural events–such as the COVID-19 pandemic–and may (not) lead to consequences that affect aspects of what individuals value (Renn and Rohrmann, 2000, p. 13). Within this process, individuals receive signals (such as lockdowns and a threatening labour market crisis), as well as information about possible future outcomes (e.g., job and income loss) and then tend to form respective opinions and attitudes toward the impact. Thus, risk perception can be defined as individual’s evaluation of possible outcomes they are or will be exposed to Taylor-Gooby and Zinn (2006) and Lidskog and Sundqvist (2013).

With respect to prior research focusing on the COVID-19 pandemic, it has been shown, that due to higher health risks, confinement-related adjustments in daily routines, a reduction of social contacts outside the household, additional screen-time and fewer opportunities for physical (outdoor) activities, risk perceptions are deteriorating (Prime et al., 2020; Möhring et al., 2021). Amongst other things, this applies to growing socioeconomic insecurities (e.g., because auf *Kurzarbeit* (short-term), layoffs or income loss) as well as, in turn, worsening individuals’ personal assessment about their future living standards. All parents (Hipp and Bünning, 2021; Li et al., 2022), but in particular single parents were challenged, since they had to manage the double burden of paid employment and additional care work at the same time (Bariola and Collins, 2021). In line with this, Calvano et al. (2022) and Racine et al. (2021) suggest that managing child care obligations, employment assignments and complying with the confinement measures was one main contributing factor for the decline in parents’ mental health. Against this background, we assume that single-parent households show worse subjective risk perceptions, compared to other household types during the COVID-19 pandemic, since they are disadvantaged by not having a partner to rely on emotionally or economically in times of crisis (Hypothesis 1).

Furthermore, prior research reveals that next to the household type other individual characteristics have an impact on risk assessments during the COVID-19 crisis, such as age, education, migration background, or employment status. For example, Wanberg et al. (2020) displays that highly educated individuals experience a greater increase in depressive symptoms and a greater decrease in life satisfaction from before to during COVID-19 in comparison to those with lower education. Kivi et al. (2021) reveal that although senior adults aged 65–71 perceived high societal risks related to the pandemic, the majority was neither particularly worried about their financial situation nor showed pronounced declines in their overall well-being. Finally, there is research emphasising the disproportionately harsh impact on unprivileged populations such as migrants. These populations are often more exposed to infections, but less protected, while at the same time being at higher risk of suffering from poor living and working conditions, and limited access to healthcare, all of which is challenging to their mental health (Garrido et al., 2023). Bearing those results in mind, we account for heterogeneity in the individual risk perception by including control variables, such as age, gender, education, migration background and employment status.

### 3.2 Objective indicator: equivalised income

As the objective indicator we consider equivalised earnings derived from the disposable household income of each individual’s household. Here, single-parent households comprise a vulnerable group on the labour market, facing above average financial hardship (Gornick and Meyers, 2003; Wu and Eamon, 2011; Maldonado and Nieuwenhuis, 2015; Nieuwenhuis and Maldonado, 2018). Per definition, they not only lack a second parent but also a second (potential) earner in the household. Furthermore, their income is a reflection of disadvantaged labour market positions due to avoidance of jobs, which require long working hours or overtime hours, and instead choosing jobs which offer flexible working arrangements but come with lower earnings (Casey and Maldonado, 2012). Thus, single parents face a double burden as they are likely to have a deficit in both money and time, with less money to pay for professional childcare and fewer hours during the day to work and care for their children (Nieuwenhuis and Maldonado, 2018, p. 172).

The pandemic added additional fuel to this already tense situation (Cook and Grimshaw, 2021). First, single parents in Germany have an above-average employment rate within the service industry (e.g., gastronomy, trading sector), which usually offers flexible working arrangements necessary for balancing the work–family conflict. For example, in 2020, more than 17% of single-parent households were employed in the trading industry compared to 12% of couple-parent households and 9% of singles without children (GSOEP 2020/21; own calculation). However, during the pandemic large parts of the service sector were shut down for many months, either forcing employees to work in *Kurzarbeit* (short-time) and reduced wages or even facing layoffs. Second, the pandemic drastically changed daily working routines and the way in which work was done. Here, working (remotely) in paid employment (from home), combined with an additional burden of unpaid care work, was difficult or even impossible for single parents, hence lowering their labour productivity. In this light, we expect that single-parent households continue to be worse off and earn less than other household types during the pandemic (Hypotheses 2).

### 3.3 Toward an understanding of the link between household type, risk perception, and income

The central aim of this paper is to investigate the relationship between household type, risk perception, and income. However, there are two ways to look at this relationship, each embedded in another strand of existing research. On the one side, we find a growing body of research concentrating on single-parent households, especially on single mothers, and their well-being or life satisfaction (Branowska-Rataj et al., 2014; Ifcher and Zarghamee, 2014; Pollmann-Schult, 2018). This life satisfaction penalty for single mothers is commonly attributed to elevated emotional and financial stress, high levels of role overload, time pressure, and strain that accompany long-term single parenting (Nelson et al., 2013; Pollmann-Schult, 2018). These studies find that although single mothers are substantially less happy than individuals in other household types, their happiness increased in absolute and relative terms over the past few decades (Herbst, 2012); here Ifcher and Zarghamee (2014, p. 1234) suggest some “possible explanations for the observed trends: changes to social welfare programs, increased labor force participation, compositional shifts in single motherhood, and reduced stigma.” Within this strand of research, objective indicators (such as income for example) are either used as control variables, or to explain differences within the group of single-parent households.

On the other hand, there is research which has established a link between objective indicators (e.g., material goods and resources like income or wealth) and subjective indicators (e.g., risk perception, well-being, life satisfaction or happiness) (Cummins, 2000; Lever, 2004; Cho, 2018; Riederer et al., 2021; Fritsch et al., 2023). This line of research indicates that the material conditions of life are related to and constitute a reliable predictor of the individual assessments of one’s life (Burchell, 2011; Clark et al., 2013; Van der Meer, 2014). However, findings on the concrete direction of this relationship remain controversial. In short, some scholars sustain a strong positive relationship, where rich people are happier with their lives, and this relationship is more pronounced, the richer the individuals are (Esterlin, 2001; Lever, 2004). Others question this relationship, affirming that a significant part of the variance of one’s subjective assessment is not directly explained by economic variables, but rather by other psychological and physiological variables–themselves contributing a significant influence (Fuentes and Rojas, 2001; Diener and Biswas-Diener, 2002).

**Figure 1:**
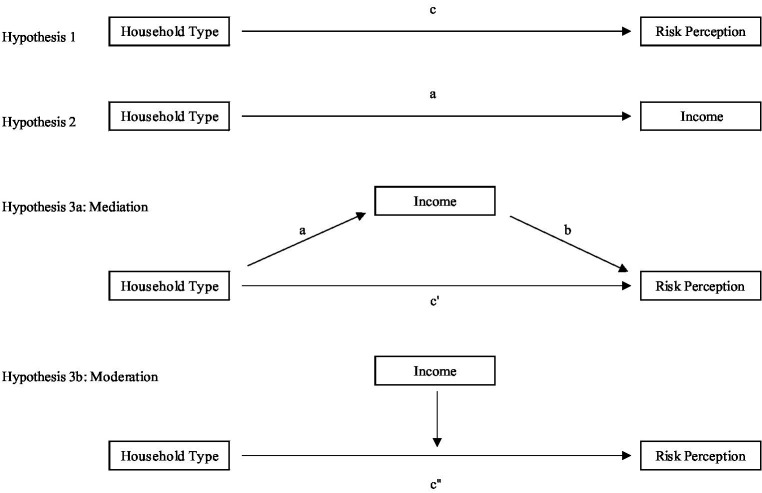
Disentangling the link between household type, risk perception and income. Source: own illustration.

In order to contribute to the current state of research, in the present paper, we argue that the relation between risk perception (subjective indicator), income (objective indicator) and household type, is anything but straightforward. We analyse this seemingly obvious relationship by dismantling the underlaying mechanisms step by step. From an analytical perspective two main mechanisms are plausible, which could influence the effect of household type on risk perception (see Figure 1). First, a *mediating effect*, where an independent variable influences a dependent variable through a third–the mediating–variable which is related to both the independent and the dependent variable (Baron and Kenny, 1986; Cho, 2018). With respect to our research focus, this would mean that the difference in risk perception of single-parent households can be explained through a third indicator, namely income. This mediating effect will reveal, in the path model, once we look at the relationship of household type, risk perception and income at the same time, that income (partially) accounts for the link between household type and risk perception. Considering that single-parent households are more likely to face financial hardship and are above average confronted with unstable labour market conditions during the COVID-19 pandemic, it seems plausible that the economic component contributes some explanatory power for the different level of risk perception of single-parent households in comparison to other household types (Hypothesis 3a). Second, we expect to observe a *moderating effect*, where the third variable alters the direction or strength of the relationship between an independent variable and a dependent variable (Baron and Kenny, 1986). In our case, this would mean that the level of income affects the relationship of household type and risk perception, revealing different levels of risk perception of household types across the earnings distribution. Considering the concrete position at the income level is particularly important since financial conditions may change massively during a crisis and have shown to be a substantial predictor for risk perceptions in times of uncertainty (Burns et al., 2012). Since single-parent cannot balance financial hardship or income loss with the help of a second adult earner in the household (Eibach and Moch, 2011), the potential consequences of low(er) incomes may weigh somewhat stronger for this special risk group. Against this background, we assume comparatively less negative risk perceptions regardless of the household type at the upper end of the income distribution, while at the lower end we expect increased negative risk perceptions amongst parents and especially amongst single parents who lack the support of a partner (Hypothesis 3b).

## 4 Data, methods and variables

### 4.1 Data

As our empirical basis we use the harmonized data from the sub-survey of the German Socio-Economic Panel, the SOEP-CoV sample,^5^ which contains details on specific household circumstances during the pandemic, including objective information on the household economic conditions, as well as subjective assessments of the current and future situation. The initial sample consist of 8,133 individuals; once we consider valid information on our main variables of interest (risk perception, household type, income, and controls) our final sample contains information on 6,065 respondents (2,502 men and 3,563 women).^6^ We restrict our sample to 2020 since important information on households–including household type and individual incomes–are not available for 2021.

### 4.2 Analytical strategy and variables

For our analytical strategy, we use a three-step procedure. In the first two steps, we are interested in how individuals living in single-parent households assess their situation during the pandemic whilst examining the subjective indicator of risk perception and the objective indicator of income. Throughout our modelling strategy, we compare individuals living in single-parent households to individuals living in three other household types [(1) singles without children, (2) couple-parent households with children, and (3) couples without children].^7^ For measuring the subjective indicator of individual risk perception respondents are asked (a) how likely they think that their living standards will diminish due to the pandemic (from 0 to 100%) or (b) if it already happened, enter 1, which we translate into 100% likelihood, since they already see the pandemic diminish their living standards.^8^ As the objective indicator, we use the logarithmised equivalised household income^9^ of individuals. In the first two steps we calculate linear regressions and present unstandardized coefficients for the subjective and objective indicator (Table 2).^10^

**Table 2:** Linear regression modelling (unstandardized coefficients, incl. controls).

	Hypothesis 1	Hypothesis 2	
	Risk perception (subjective indicator)	Income (objective indicator)	Risk perception (subjective indicator)
Income	−	−	−3.56***
Household type			
Single-parent households	Ref.	Ref.	−
Singles without children	−3.14**	0.15***	−
Couples without children	−5.08***	0.38***	−
Couple-parent households	−2.08+	0.16***	−
Controls			
Female	1.33+	0.04***	1.77***
Age	−0.22***	0.01***	−0.21**
Migration background	5.73***	−0.015***	5.30***
Education			
Low	Ref.	Ref.	Ref.
Middle	−0.63	0.19***	−0.09
High	−1.92**	0.43***	−0.59
Employment status			
Full-time	Ref.	Ref.	Ref.
Part-time	2.40*	−0.22***	1.78+
Unemployed	10.65***	−0.58***	8.71***
Non-employed	0.70	−0.40***	−1.09
*N*	6,065	6,065	6,065

Source: CoV-Sample 2020; own calculation, +*p* < 0.10, *p < 0.05, **p < 0.01, ***p < 0.001.

The dependent variable in Model 1 is the subjective indicator of individual risk perception of worsening living standards; the dependent variable in Model 2 is the objective indicator of logarithmised equivalised disposable household income; the dependent variable in Model 3 is individual risk perception of worsening living standards.

**Figure 2:**
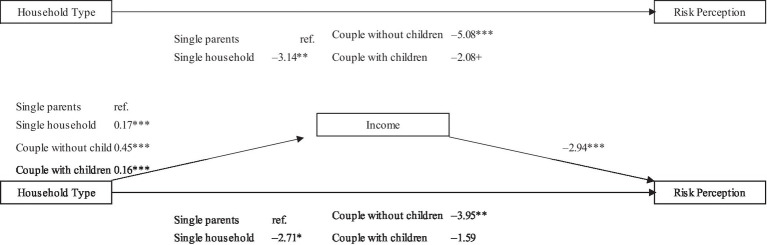
Path model: disentangling the link between household type, risk perception and income (unstandardized coefficients, incl. controls). Source: CoV-Sample 2020; own calculations, +*p* < 0.10, *p < 0.05, **p < 0.01, ***p < 0.001. As subjective indicator we use individual risk perception of worsening living standards; as objective indicator we use logarithmised equivalised disposable household income; path modelling controls for gender, age, migration background, education, employment status, kids in school, kids in pre-school.

**Figure 3:**
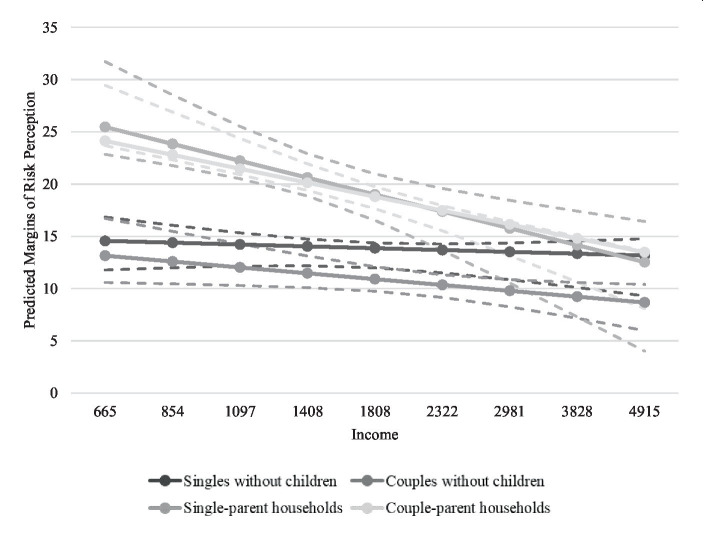
Moderation effect of income (Hypotheses 3b) (incl. controls). Source: CoV-Sample 2020; own calculations. As subjective indicator we use individual risk perception of worsening living standards; as objective indicator we use logarithmised equivalised disposable household income (axis labels in Euros).

In the next step we estimate a path model to uncover a possible mediation effect of income intervening in the risk perception of different household types (Aichholzer, 2017, p. 51; Baron and Kenny, 1986) and an interaction model between income level and household type to test whether there is a moderation effect of income, meaning that the effect of household type differs across different income levels (results are displayed in Figures 2, 3). According to our analytical strategy, we are interested in how earnings mediate and moderate the effect of household type on economic risk perception during the COVID-19 crisis. By using logarithmised incomes, we take into account that an increase in income has a stronger effect on risk perception in lower income groups than in higher income groups. As differences in the compositions of the household groups might be present in relation to other variables might affect risk perceptions directly, control variables we include are *gender* as a dummy variable (0 = men, 1 = women), *age* as a metric variable, *migrant background* as a dummy variable (0 = no migration background, 1 = direct or indirect migration background^11^), level of *education* (low, mid: vocational, and high: university), *employment status* (full-time, part-time, marginal, short work, unemployed or non-employed).^12^

## 5 Results

### 5.1 How do individuals living in single-parent households make it through the COVID-19 pandemic and how do they assess their future?

To evaluate how individuals living in different household types muddle through during the COVID-19 crisis, and whether single-parent households are particularly at risk concerning their future, we first contrast individuals’ present economic situation as well as their subjective evaluation of their prospects. Table 2 displays differences in risk perceptions (subjective indicator) and income (objective indicator) between individuals living in different household types, based on linear regression modelling after controlling for sociodemographic variables. A set of relevant findings result from these models: With respect to the subjective indicator of risk perceptions (Table 2, column 1), we observe that couples without kids and individuals living in couple-parent households assess their situation less negatively during the pandemic, compared to individuals living in single-parent households and single households (Hypothesis 1).^13^ Thus, our findings confirm prior research addressing household type as well as marital status as specifically important when it comes to detrimental consequences during the COVID-19 crisis (Reichelt et al., 2020; Bariola and Collins, 2021; Hiekel and Kühn, 2022). Moreover, looking at the control variables in Model 1, we can conclude that future prospects are rated less negatively with increasing age (Kivi et al., 2021) and worse if respondents have direct or indirect migration background (Garrido et al., 2023). Furthermore, the individual evaluation of future living standards is less negative the better educated and worse if individuals are currently unemployed or working part-time.

With respect to the objective indicator (Table 2, column 2) we notice that individuals living in single-parent households have to face a worse situation compared to individuals living in all other household types (Hypothesis 2). The unstandardized coefficients indicate that the gap in disposable household income is highest between individuals living in single-parent households and couples without children; but couples with children and singles are financially better off as well. Concerning our control variables Model 2 reveals patterns commonly known for Germany and other corporatist welfare states (Esping-Andersen, 1990; Orloff, 1996; Teitzer et al., 2014): We observe that the financial situation improves with increasing age, and for highly educated individuals. However, it deteriorates for individuals with migrant background, in flexible employment relationships–such as part-time jobs–, as well for unemployed or economically inactive individuals.

Against this background and in line with prior research (Hipp and Bünning, 2021; Li et al., 2022), our findings affirm our first and second assumption. We observe that individuals living in single-parent households have detrimental future perspectives and are worse off with respect to their economic position compared to individuals living in other household types. Finally, in Model 3 (Table 2, column 3) we account for the effect of income on subjective risk perception, which shows a negative association. This means, that individuals with a lower household income estimate a higher probability of a worsened living standards due to the COVID-19 crisis. Looking at the bigger picture, our findings suggest that having a low income, which might in turn be related to unstable and precarious labour market situations, is likely to reduce the probability of positive feelings, including exerting environmental control, and in projecting oneself into a brighter future (Cummins, 2000, p. 138).

### 5.2 How does income affect the subjective risk perception of individuals living in single-parent households during the pandemic?

In Figure 2 we present the results of our analyses displaying the link of household type, risk perception and income. Here, we are interested in whether or not individuals living in single-parent households evaluate their situation during the pandemic more negatively because they are single-parents, or rather because they are financially worse off. Here, we used path modelling in order to account for the relationship between the subjective indicator of risk perception, the objective indicator of earnings and household type. In the upper part of Figure 2, we again display that couples without kids and couple-parent households assess their situation during the pandemic less negatively compared to single-parent households and single households. In the next step, we include income into the relationship between household type and risk perception as a mediating variable; our findings reveal that the direct effect of household type on risk perception is in part not statistically significant anymore. Put differently, the direct effect of household type on subjective risk perception partly disappears (Table 3).

**Table 3:** Path model (unstandardized coefficients).

	Hypothesis 3a: path model (direct effects)			
	Model I		Model II (incl. controls)	
	Income	Risk perception	Income	Risk perception
Income	−	−6.62***	−	−2.94***
Household type				
Single-parent households	Ref.	Ref.	Ref.	Ref.
Singles without children	0.17***	−5.62***	0.17***	−2.71*
Couples without children	0.45***	−7.19***	0.45***	−3.95**
Couple-parent households	0.16***	−0.27	0.16***	−1.59
Controls				
Female	−	−	−	1.46*
Age	−	−	−	−0.20***
Migration background	−	−	−	5.29***
Education				
Low	−	−	−	Ref.
Middle	−	−	−	−0.08
High	−	−	−	−0.67
Employment status				
Full-time	−	−	−	Ref.
Part-time	−	−	−	1.75+
Unemployed	−	−	−	8.95***
Non-employed	−	−	−	−0.47
*N*		6,065		6,065

Source: CoV-Sample 2020; own calculations, +*p* < 0.10, *p < 0.05, **p < 0.01, ***p < 0.001.

As subjective indicator we use individual risk perception of worsening living standards; as objective indicator we use logarithmised equivalised disposable household income.

In more detail, path modelling reveals statistically significant differences in the average income of individuals living in different household types–singles, couples with children and especially couple-parent households earn more compared to single-parent households. Furthermore, the significant indirect effects of household type on risk perception via income indicate that income is an important mediator for a family’s evaluation of their future standard of living (Hypothesis 3a).

With respect to our last hypothesis (3b), we also observe a statistically significant moderating effect of income on the risk perception by household type. Individuals living in childless households with lower income show significantly less negative risk perceptions than households with children (Figure 3 and Table A2 in Supplementary material). Moreover, while income does not play a role in the risk perception of single and couple households without children, for couple households with children as well as individuals living in single-parent households a lower income increases their risk perception. This increase in risk perception is significantly stronger for individuals living in single-parent households than for individuals living in households without children. We find no significant differences, however, between individuals living in single-parent households and individuals living in couple-parent households. The interaction of household type and income reveals that income moderates the perceptions of those who live with children, regardless of whether they have a partner or not. Thus, we can see that in fact households with children that do have a high income also do not suffer from a more negative risk perception in comparison to other household types (Hypothesis 3b, only partially confirmed – no differences between single and couples parents).^14^

Against this background, and in line with other research, our findings show that a significant part of the variance of one’s subjective assessment can be directly explained by economic conditions (e.g., income or wealth) (Esterlin, 2001; Lever, 2004; Burchell, 2011; Clark et al., 2013; Van der Meer, 2014). Thus, we conclude that the effect of household type on risk perception is mediated and moderated via the household income. All in all, we conclude that single-parent households are not worse off *per se* during COVID-19 pandemic. However, based on the SOEP-CoV data for 2020 for Germany, our results reveal that the risk perception of individuals living in single-parent households is worse on average because of their financially vulnerability.

## 6 Discussion

The COVID-19 pandemic had severe consequences on the lives of millions of individuals around the globe. However, some have been hit harder than others: For sure, individuals living in single-parent households account for a vulnerable group, especially and heavily at risk of facing financial distress and emotional hardship. In the paper at hand, we put the situation of individuals living in single-parent households during the pandemic in Germany at centre-stage. By focusing on the relationship of subjective and objective measures of financial and emotional struggles we show how they are intertwined. We started by displaying a historical perspective on the economic situation of individuals living in single-parents households, whilst comparing them to individuals living in other household types. This descriptive time series highlights the consistently exposed position of individuals living in single-parent households over two decades and points toward a recent widening of pre-existing social trenches among different societal groups. After setting the scene for single-parent households’ circumstances of life in the years before the crisis began, we applied a three-step analytical procedure in order to disentangle the relationship of household type, risk perception and incomes during the pandemic.

Based on the SOEP-CoV data for 2020 for Germany, our findings once again underline the strong financial vulnerability of individuals living in single-parent households during the COVID-19 pandemic and highlight that they are most vulnerable to worsen their perception of future living standards. Although this first set of findings might not come as a surprise, it is nonetheless a relevant finding for evidence-based social policy decisions–especially if we consider that around 6% of the households are single-parent households in Germany. Thus, our study is in line with other research, pointing toward the unequal effects of the pandemic, particularly affecting those who were already in precarious situations (Wachtler et al., 2020; Kuhn et al., 2021). We add to the body of literature addressing the pronounced increase in inequality and growing economic risks for individuals living in different household types (Huebener et al., 2021). In this respect, we specifically refer to Schäfermayer et al. (2022), who likewise showed that single parents worried more than couple-parents in partnerships largely due to their bleak socio-economic conditions. Furthermore, our second set of findings is new and contributes to the current literature by clarifying the entangled relationship between household type, objective indicators (such as material goods, income or wealth) and subjective indicators (such as risk perception, well-being or happiness) (Cummins, 2000; Lever, 2004; Clark et al., 2013; Cho, 2018). We used path and interaction models to show that earnings mediate and moderate the effect of household type on economic risk perception during the COVID-19 crisis. These findings indicate that individuals living in single-parent households do not perceive higher risks of worsening living standards due to their household situation *per se*, but rather because they are worse off in their economic situation compared to individuals living in other household types. In our view, this is a relevant finding. Although individuals living in single-parent households are in need of more support and are worse off during the pandemic in Germany, our results could nevertheless serve as tiny ray of hope. Governmental support programs are not set out to change the household structure. However, they are very well able and all the more urged to improve the currently poor income situation of this vulnerable group.

Finally, our study is limited since we were not able to consider the full spectrum of objective and subjective risks faced by individuals living in single-parent households in the past 2 years. Next to the individual assessment of worsening living standards in the present/future and income, there are plenty of other indicators which can be used to describe the situation during the pandemic. Furthermore, we did not analyse the evaluation of present situation of single-parent households, but rather their prospects. Finally, longitudinal analysis is needed to further disentangle the layered relationship of subjective and objective indicators and to uncover the causal effect of the pandemic on the different household types.

## Data availability statement

The data analyzed in this study is subject to the following licenses/restrictions: You have to purchase the data. Requests to access these datasets should be directed to https://www.diw.de/en.

## Author contributions

BL: Conceptualization, Methodology, Visualization, Writing – original draft, Writing – review & editing. N-SF: Conceptualization, Writing – original draft, Writing – review & editing, Methodology. CSM: Conceptualization, Formal analysis, Methodology, Writing – original draft, Writing - review & editing. RV: Supervision, Writing – original draft.

## Footnotes

^1^We use the term single-parent households to refer to individuals living in households, declaring themselves as single parents. This includes single parents (mothers or fathers) who raise one or more children living in the same household, while not living in the same household with another adult (e.g., their partner, grandparents), or (currently) not having a partner. By using this definition, we do not differentiate between parents who were single when they had their child and those who got separated afterwards or were bereaved (Nieuwenhuis and Maldonado, 2019). By comparison, we refer to couple-parent households to reflect that both adults are living in the same household and are parenting one or more children also living in this household. Singles per definition do not have children and do not share their living environment with another adult. Finally, we define couples without children as two adults living in the same household.

^2^In 2020, compared to the population average (poverty rate: 16 percent) and in relation to individuals living in couple-parent households with children (poverty rate: 12 percent) single-parent households share the highest poverty rates (31 percent), which accounts for an increase of almost seven percentage points in two decades.

^3^In Germany, inflation rates were mostly low (max 2% annually) until 2021 (Statistisches Bundesamt, 2021). Since for our empirical analysis we are analysing data until 2020, inflation should not affect the quality of our results.

^4^Although referring to desirable risks which individuals aspire to reach, rather than relating to the danger of unwanted events, is per definition plausible as well (Machlis and Rosa, 1990), but not subject to this paper.

^5^The sub-survey was dedicated to monitor the pandemic situation. A total of 12,000 households were asked to participate in the SOEP-CoV study. The first wave of the survey started on April 1, 2020, and ended on June 28, 2020. Individuals from a total of 6,694 households were surveyed.

^6^Compared to the original sample, we excluded about 761 respondents not living in one of the four household types we are interested in and 32 respondents because of their age. In addition, 1,275 respondents do have missing values for one of the other variables.

^7^We use single-parent households as reference category to compare this group with all other household types in the regression models.

^8^For sensitivity analyses, we additionally calculated two models with (a) the metric variable assessing the likelihood that the living standard will diminish due to the pandemic excluding those with already lower living standard; and (b) the binary variable that the living standard already diminished or not. For (a) the results show the very same patterns of mediation and moderation like the models presented in section 5. For (b) the patterns of mediation are still the same, however, the interaction effect of household type and income is not significant for this model.

^9^The equivalised disposable income is defined as the total disposable income of a household, divided by the equivalised number of household members; household members are weighting each according to their age, using the modified OECD equivalence scale (Eurostat, 2021). In order to reflect differences in a household’s size and composition, the total disposable household income is divided by the number of ‘equivalent’ individuals (1.0 to the first adult; 0.5 to the second and each subsequent person aged 14 and over; 0.3 to each child aged under 14) (Eurostat, 2021).

^10^In order to substantiate our findings, we calculated a number of sensitivity analyses. For the models in Table 2, we additionally calculated linear regressions without control variables, which are presented in the supplementary material Table A1.

^11^Being born in another country than Germany indicates, by definition, a direct migration background, while respondents born in Germany may have either no or an indirect migration background. Respondents whose parents had no migration background were assigned the code “no migration background.”

^12^For sensitivity analyses, we additionally calculated our models with variables including the age of the children (e.g., “kids in school” and “kids in preschool”) and employment security, accounting for (1) more time-consuming parenting work and possibly more worries about the development of their children during the pandemic and (2) occupation and type of contract. The results show that parents were in general more worried and being in a partnership does not seem to moderate this risk perception. With respect to the age of children, there does seem to be an independent effect of having young children (beneath age 6) on making the risk perception of individuals more negative. When we add these variables to the models, they explain part of the differences between household types. However, we did not include the age of the children into our final models since we already account for children in the definition of the household types. When we additionally consider employment security the main results remain stable (see Tables A3 and A4 in the Supplementary material).

^13^Since the dependent variable measures worsening living standards, a negative sign translates into a less negative assessment.

^14^To substantiate our findings, we calculated a number of sensitivity analyses. We included individual health status and source of information on COVID-19 (e.g., watching the news, reading newspapers) into the path model in order to evaluate, whether for example high stress levels of individuals living in single-parent households or a poor health status is influencing the relationship between household type, income and risk perception. These sensitivity analyses reveal that neither do individuals living in single-parent households show a worse health status, nor is the individual health status related to the risk perception. We further find that individuals living in single-parent households use different information sources than individuals living in other household types. However, even if we include the source of information into our model the initial relationship between household type, income and risk perception does not change.
