# Supplementary material for: Risk perceptions of individuals living in single-parent households during the COVID-19 crisis: examining the mediating and moderating role of income
Source: Front Sociol. 2023 Nov 30;8:1265302. doi: 10.3389/fsoc.2023.1265302 (PMC10746366; doi:10.3389/fsoc.2023.1265302)
Supplement: Supplementary file 1 [file Table_1.docx]

Risk Perceptions of Individuals living in Single-Parent Households during the COVID-19 Crisis: Examining the Mediating and Moderating Role of Income

**Supplementary Material**

**Table A1: Linear regression modelling** (unstandardized coefficients, no controls)

|  | Hypothesis | 1 | Hypothesis 2 |  |  |  |
| --- | --- | --- | --- | --- | --- | --- |
|  | Risk perception | | Income |  | Risk perception | |
|  | (subjective indicator) | | (objective indicator) | | (subjective indicator) | |
| Income | - |  | - |  | -8.02 | *** |
| Household type |  |  |  |  |  |  |
| Single-parent households | ref. |  | ref. |  | - |  |
| Singles without children | -6.74 | *** | 0.17 | *** | - |  |
| Couples without children | -10.18 | *** | 0.46 | *** | - |  |
| Couple-parent households | -1.32 |  | 0.16 | *** | - |  |
| N | 6,065 |  | 6,065 |  | 6,065 |  |

Source: CoV-Sample 2020; own calculations, +p < 0.10, *p < 0.05, **p < 0.01, ***p < 0.001.

Note: The dependent variable in Model 1 is the subjective indicator of individual risk perception of worsening living standards; the dependent variable in Model 2 is the objective indicator of logarithmised equivalised disposable household income; the dependent variable in Model 3 is individual risk perception of worsening living standards.

**Table A2: Moderation effect of income (Hypothesis 3b)** (unstandardized coefficients, incl. controls)

|  | Risk Perception |  |
| --- | --- | --- |
| Income | -6.46 | ** |
| Household type |  |  |
| Single-parent households | ref. |  |
| Singles without children | -44.83 | * |
| Couples without children | -34.91 | + |
| Couple-parent households | -9.39 |  |
| Interaction term |  |  |
| Singles without children X income | 5.76 | * |
| Couples without children X income | 4.22 | + |
| Couple-parent households X income | 1.13 |  |
| Controls |  |  |
| Female | 1.53 | * |
| Age | -0.20 | *** |
| Migration background | 5.03 | *** |
| Education |  |  |
| Low | ref. |  |
| Middle | 0.15 |  |
| High | -0.38 |  |
| Employment Status |  |  |
| Full-time | ref. |  |
| Part-time | 1.86 | * |
| Unemployed | 8.70 | *** |
| Non-employed | -0.50 |  |
| N | 6,065 |  |

Source: CoV-Sample 2020; own calculations, +p < 0.10 *p < 0.05, **p < 0.01, ***p < 0.001.

Note: As subjective indicator we use individual risk perception of worsening living standards; as objective indicator we use logarithmised equivalised disposable household income.

**Table A3: Models including age of youngest child in household** (unstandardized coefficients)

|  | Hypothesis 3a | | |  | Hypothesis 3b | |
| --- | --- | --- | --- | --- | --- | --- |
|  | Income |  | Risk Perception | | Risk Perception | |
| Income | - |  | -2.71 | *** | -5.74 | * |
| Household type |  |  |  |  |  |  |
| Single-parent households | ref. |  | ref. |  |  |  |
| Singles without children | 0.17 | *** | -1.41 |  | -38.19 | * |
| Couples without children | 0.45 | *** | -2.72 | * | -28.98 |  |
| Couple-parent households | 0.16 | *** | -2.28 | + | -7.70 |  |
| Interaction term |  |  |  |  |  |  |
| Singles without children X income | - |  | - |  | 4.99 | * |
| Couples without children X income | - |  | - |  | 3.55 |  |
| Couple-parent households X income | - |  | - |  | 0.82 |  |
| Controls |  |  |  |  |  |  |
| Female | - |  | 1.49 | * | 1.54 | * |
| Age | - |  | -0.19 | *** | -0.19 | *** |
| Migration background | - |  | 5.27 | *** | 5.04 | *** |
| Education |  |  |  |  |  |  |
| Low | - |  | ref. |  |  |  |
| Middle | - |  | -0.03 |  | 0.20 |  |
| High | - |  | -0.68 |  | -0.42 |  |
| Employment Status |  |  |  |  |  |  |
| Full-time | - |  | ref. |  |  |  |
| Part-time | - |  | 1.67 | + | 1.79 | + |
| Unemployed | - |  | 8.89 | *** | 8.70 | *** |
| Non-employed | - |  | -0.50 |  | -0.47 |  |
| Kids in school | - |  | 2.39 | * | 1.96 | * |
| Kids in pre-school | - |  | 1.78 |  | 1.14 |  |
| N |  |  | 6,065 |  | 6,065 |  |

Source: CoV-Sample 2020; own calculations, +p < 0.10 *p < 0.05, **p < 0.01, ***p < 0.001.

Note: As subjective indicator we use individual risk perception of worsening living standards; as objective indicator we use logarithmised equivalised disposable household income.

**Table A4: Job security specification** (unstandardized coefficients)

|  | Hypothesis 3a | | |  | Hypothesis 3b | |
| --- | --- | --- | --- | --- | --- | --- |
|  | Income |  | Risk Perception | | Risk Perception | |
| Income | - |  | -2.70 | *** | -6.07 | ** |
| Household type |  |  |  |  |  |  |
| Single-parent households | ref. |  | ref. |  | ref. |  |
| Singles without children | 0.17 | *** | -2.46 | * | -42.52 | * |
| Couples without children | 0.45 | *** | -3.83 | ** | -33.72 | + |
| Couple-parent households | 0.16 | *** | -1.27 |  | -8.61 |  |
| Interaction term |  |  |  |  |  |  |
| Singles without children X income | - |  | - |  | 5.48 | * |
| Couples without children X income | - |  | - |  | 4.08 |  |
| Couple-parent households X income | - |  | - |  | 1.07 |  |
| Controls |  |  |  |  |  |  |
| Female | - |  | 1.63 | * | 1.70 | ** |
| Age | - |  | -0.20 | *** | -0.19 | *** |
| Migration background | - |  | 5.23 | *** | 4.99 | *** |
| Education |  |  |  |  |  |  |
| Low | - |  | ref. |  | ref. |  |
| Middle | - |  | -0.23 |  | -0.00 |  |
| High | - |  | 0.36 |  | 0.62 |  |
| Employment Status |  |  |  |  |  |  |
| ISCO 1–3 | - |  | ref. |  | ref. |  |
| ISCO 4–9 | - |  | 4.87 | *** | 4.81 | *** |
| Unemployed | - |  | 8.54 | ** | 8.17 | ** |
| Non-employed | - |  | -0.22 |  | -0.38 |  |
| Type of Contract |  |  |  |  |  |  |
| Standard contract | - |  | ref. |  | ref. |  |
| Non-standard contract | - |  | 1.09 |  | 1.19 |  |
| Not in employment | - |  | 0.89 |  | 1.01 |  |
| N |  |  | 6,065 |  | 6,065 |  |

Source: CoV-Sample 2020; own calculations, +p < 0.10 *p < 0.05, **p < 0.01, ***p < 0.001.

Note: As subjective indicator we use individual risk perception of worsening living standards; as objective indicator we use logarithmised equivalised disposable household income; non-standard contracts including part-time, marginal, short-time and temporary employment.
